# Polymorphism Analysis of *pfmdr1* and *pfcrt* from *Plasmodium falciparum* Isolates in Northwestern Nigeria Revealed the Major Markers Associated with Antimalarial Resistance

**DOI:** 10.3390/diseases9010006

**Published:** 2021-01-04

**Authors:** Ruqayya Adam, Muhammad M. Mukhtar, Umar F. Abubakar, Hajara A. Damudi, Abdullahi Muhammad, Sulaiman S. Ibrahim

**Affiliations:** 1 Department of Biological Sciences, Federal University Dutsinma, Katsina PMB 5001, Nigeria; radam@fudutsinma.edu.ng; 2 Department of Biochemistry, Bayero University, Kano PMB 3011, Nigeria; muhammadmahemukhtar@gmail.com (M.M.M.); yusufhajara72@gmail.com (H.A.D.); 3 Laboratory Department, Public Health and Diagnostic Institute, Yusuf Maitama Sule University, Kwanar Dawaki, Kano PMB 3220, Nigeria; farouqabu@nwu.edu.ng; 4 Centre for Biotechnology Research, Bayero University, Kano PMB 3011, Nigeria; Abdullahi.Muhammad@lstmed.ac.uk; 5 Liverpool School of Tropical Medicine LSTM, Pembroke Place L3 5QA, UK

**Keywords:** *Plasmodium falciparum*, *pfmdr1*, *pfcrt*, mutation, antimalarial, resistance, Nigeria

## Abstract

Suspicion of failure in the effectiveness of artemisinin-based combination therapies (currently the first-line treatment of malaria, worldwide) is leading to the unofficial use of alternative antimalarials, including chloroquine and sulfadoxine/pyrimethamine, across northern Nigeria. To facilitate evidence-based resistance management, antimalarial resistance mutations were investigated in *Plasmodium falciparum multidrug resistance-1* (*pfmdr1*) and *chloroquine resistance transporter* (*pfcrt*), in isolates from Kano, northwestern Nigeria. Out of the 88 samples genotyped for *pfmdr1* N86Y mutation using PCR/restriction fragment length polymorphism, one sample contained the 86Y mutation (86Y_frequency_ = 1.14%). The analysis of 610 bp fragments of *pfmdr1* from 16 isolates revealed two polymorphic sites and low haplotype diversity (H_d_ = 0.492), with only 86 Y mutations in one isolate, and 184 F replacements in five isolates (184F_frequency_ = 31.25%). The analysis of 267 bp fragments of *pfcrt* isolates revealed high polymorphism (H_d_ = 0.719), with six haplotypes and seven non-synonymous polymorphic sites. Eleven isolates (61.11%) were chloroquine-resistant, CQR (C_72_V_73_I_74_E_75_T_76_ haplotype), two of which had an additional mutation, D^57^E. An additional sequence was CQR, but of the C_72_V_73_M_74_E_75_T_76_ haplotype, while the rest of the sequences (33.33%) were chloroquine susceptible (C_72_V_73_M_74_N_75_K_76_ haplotype). The findings of these well characterized resistance markers should be considered when designing resistance management strategies in the northwestern Nigeria.

## 1. Introduction

Malaria killed approximately 405,000 people in 2018, of which 93% were in sub-Saharan Africa [1]. Nigeria, the country with the highest global burden of malaria, accounted for 25% of these deaths [1]. Antimalarials and vector control had significantly reduced malaria mortality in the last two decades [1,2], but antimalarial resistance (principally in *Plasmodium falciparum* which accounts for 99.7% of malaria in Africa [1]) is one of the problems stalling the progress of malaria control [3]. Prior to the emergence of resistance, chloroquine (CQ) was the effective and inexpensive drug for the treatment of non-complicated malaria [4]. The development of resistance to CQ and other antimalarial drugs, e.g., sulfadoxine/pyrimethamine (SP) in *P. falciparum* led the World Health Organization (WHO) recommending artemisinin-based combination therapy (ACT) as first line treatment for uncomplicated malaria, with more than 80 countries adopting this medicine [5]. However, the emergence of multidrug resistance, especially to artemisinin and its partner drugs, first described in western Cambodia [5], and the independent emergence of artemisinin resistance in multiple locations in the Greater Mekong Sub-region (GMS) [6,7] is threatening the efficacy of ACTs. ACT resistance is described across Africa but the major mutations linked to delayed parasite clearance and artemisinin resistance in the *pfkelch13* gene, e.g., Y493H, C580Y and Y493H, have been found only in very low frequency across Africa [8]. Indeed, a recent metadata analysis by the WorldWide Antimalaria Resistance Network (WWARN) has reported that *kelch13* mutations in African sites remained at very low prevalence, generally below 3%, and there is still no evidence of slow-clearing parasites or selection for mutant parasites [9]. This suggests that in Africa, different resistance mechanisms could be responsible for artemisinin resistance. For example, the *P. falciparum* multidrug resistance-1 transporter (*pfmdr*1), shown to be involved in the modulation of resistance/susceptibility to several antimalarial drugs [10] and the implementation of ACT shown to select for *pfcrt* mutations, and treatment failure with the ACT partner drug amodiaquine [11,12,13].

Nigeria adopted ACTs as a first-line treatment of uncomplicated *P. falciparum* in 2005, following studies that established high efficacies for artemether–lumefantrine and artesunate–amodiaquine [14]. Since, a handful of studies have documented treatment failure in patients treated with artemether and/or ACT partner drugs like amodiaquine, e.g., [13,15,16,17]. Studies carried out to detect mutations in the *kelch13* gene to date have found none of the major mutations associated with delayed parasite clearance in isolates from Nigeria [18,19,20,21,22]. However, except for [22] these recent studies were all carried out in southern Nigeria, with little known of *kelch13* resistance markers in northern Nigeria. Indeed, in the above recent study [22] none of the major mutations associated with delayed parasite clearance were found, though six other mutations were seen in the field isolates, including E^433^G and E^688^K, which were found in isolates from individuals which failed to clear infection, on 14 day of the follow-up treatment with Cartef^®^ (GB PHARMA, London, UK), an artemether/lumefantrine (80 mg/480 mg taken twice daily).

Meanwhile, a decline in the sensitivity of *P. falciparum* to ACTs has also prompted the unofficial use of CQ for self-medication in northern Nigeria [23] despite reports of 76 T *pfcrt* mutations from several studies [13,23,24,25]. It is therefore important to continue to monitor for the presence of markers known to confer CQ resistance, in view of the possible re-introduction of CQ for malaria treatment; in addition to investigating markers (e.g., N86Y mutation) in *pfmdr1* associated with decreased susceptibility to CQ and artemisinin partner drug, amodiaquine [26,27,28].

Out of the various *pfmdr1* single-nucleotide polymorphisms (SNPs) reported to date, N86Y, Y184F, S1034C, N1042D and D1246Y are the most common [29]. While NH_2_-terminal mutations (N86Y and Y184F) are more commonly found in Asian and African parasites, the COOH-terminal mutations (S1034C, N1042D and D1246Y) are encountered more often in South American isolates [28]. However, the D1246Y mutation is also present in approximately 0.7–3% of the 1502 African genomes sequenced recently [10,30,31]. The 86 Y and 184 F replacements have been reported in several studies, the majority of which were conducted in southwest Nigeria [23,24,25,30], with 86 Y and 184 F mutations linked to amodiaquine, CQ and artemether resistance [13,15,19].

Several mutations in the *pfcrt* have been linked with chloroquine-resistant (CQR) [31,32], the majority of these occurring at codons 74, 75 and 76 [33,34], with 76 T replacement (K76 T) implicated as the most important mutation [4,34,35]. The cluster of mutation in positions 72–76 has given rise to the evolution of three distinct genotypic alleles: the ancestral CQ susceptible haplotype (chloroquine-sensitive isolates (CQS), known as C_72_V_73_M_74_N_75_K_76_), and the evolved CQR haplotypes (C_72_V_73_I_74_E_75_T_76_ and S_72_V_73_M_74_N_75_T_76_) of which the former is commonly found in Asia and Africa, and the latter in the Americas [31,36,37]. Several studies in Nigeria have established the presence of *pfcrt* mutations associated with chloroquine resistance. The CVIET haplotype has been described as present in high frequency in several studies conducted in southern Nigeria [26,27,38].

To contribute to malaria control efforts in northern Nigeria, major mutations linked to antimalarial resistance in the *pfcrt* and *pfmdr1* were investigated in circulating *P. falciparum* isolates from Kano, northwestern Nigeria. The CQR haplotype CVIET and the two major mutations *pfmdr*186 Y and -184 F previously linked to multidrug resistance were all discovered, indicating the need for resistance marker surveillance to guide evidence-based resistance management and policy making.

## 2. Materials and Methods

### 2.1. Study Site, Samples and Ethics

Samples used for this study were described in a recently published work [22]. Febrile patients (18–56 years) presenting with uncomplicated malaria were recruited at Murtala Muhammad Specialist Hospital, located in Kano City, northwestern Nigeria, as described in the above study. The collection of samples (in August 2018), exclusion criteria (pregnant women and individuals who had taken antimalarial chemotherapy or prophylaxis), ethical clearance (MOH/Off/797/T.I./402) as well as written or thumb-printed consent were all described in the above study [22].

### 2.2. DNA Extraction and Confirmation of P. falciparum Infection Using PCR

Ninety-one (91) samples positive with *P. falciparum*, using the Giemsa thick and thin smear staining microscopy, as well as *cytochrome oxidase* I PCR [22] were utilized for this study. These comprised 49 samples from the above study [22], of which 11 were from individuals who failed to clear *Plasmodium* infection following 14 day treatment with Cartef. In [22], none of the major mutations known to cause delayed parasite clearance/artemisinin resistance were discovered in the *kelch13*, though six other mutations were found (including E^433^G and E^688^K, which were found in isolates from individuals who failed to clear infection, on 14 day follow-up). Genomic DNA (gDNA) was extracted from whole blood using the QIAamp^®^ DNA Mini Kit (Qiagen, Hilden, Germany) according to the manufacturer’s instruction. The DNA was eluted in 100 µL of nuclease-free water, its concentration measured using Qubit 4.0 fluorometer (Invitrogen, Waltham, MA, USA) and the samples were stored at −20 °C. A single step PCR targeting the *cytochrome oxidase* III gene [36] was used to confirm *P. falciparum* infection. One microliter (1 μL) of gDNA was added to 14 μL premix, containing 0.24 μL of dNTPs (0.4 mM), 0.375 μL of MgCl_2_ (0.63 mM), 0.2 μL (0.05 U) of KappaTaq (Kappa Biosystems, Wilmington, MA, USA), 10.66 μL of ddH_2_0, 1.5 μL of 10× TaqA buffer and 0.5 μL of primers (0.4 μM): shortCOXIII F(5′-AGCGGTTAACCTTTCTTTTT CCTTACG-3′) and shortCOXIII R (3′- AGTGCATCATGTATGACAGCATGTTTACA-5′). Thermocycling conditions were 95 °C for 3 min, followed by 35 cycles each of 94 °C for 1 min, 65 °C for 1 min, and 72 °C for 1 min; and 72 °C final extension for 10 min. The DNA of the fully susceptible *P. falciparum 3D7* provided by Dr. Janet Storm of Parasitology Department, Liverpool School of Tropical Medicine (LSTM), UK, was used as a control. PCR products were separated on 1.5% agarose gel stained with a pEqGREEN and visualized for bands.

### 2.3. Amplification of Pfmdr1 Fragment Encompassing the 86th Codon

A fragment from the *pfmdr1* encompassing the 86th codon was amplified successfully from 90 DNA samples. The nested protocol used was as described by Shrivastava et al. [4]. For first round PCR, 0.5 μL of forward and reverse primers (P1: 5′-ATGGGTAAAGAGCAGAAAGA-3′ and P2: 5′-AACGCAAGTAATACATAAAGTCA-3′), 5 μL Gotaq Green master mix (Promega, Madison, WI, USA) and 3 μL of nuclease-free H_2_O were mixed on ice. One microliter (1 μL) of gDNA was added to a final volume of 10 μL. Thermocycling conditions were 95 °C for 1 min, followed by 35 cycles each of 94 °C for 30 s, 50 °C for 30 s, 65 °C for 2 min and a 65 °C final extension for 5 min. PCR products were separated on 1.5% agarose gel stained with a pEqGREEN and visualized for bands.

For the nested PCR, 1 μL each of primers P3 (5-TGGTAACCTCAGTATCAAAGAA-3) and P4 (5-ATAAACCTAAAAAGGAACTGG-3) were mixed with 10 μL of Gotaq Green master mix and 6 μL of nuclease free H_2_O. Two microliters (2 μL) of PCR product from nest 1 were added to give a total reaction volume of 20 μL. PCR conditions were 95 °C for 1 min, followed by 35 cycles each of 94 °C for 30 s, 50 °C for 30 s, 65 °C for 1 min and 65 °C final extension for 5 min. PCR products were separated on 1.5% agarose gel stained with a pEqGREEN, and visualized for bands. These amplicons were purified using QIAquick^®^ PCR Purification Kit (Qiagen, Hilden, Germany) according to the manufacturer’s protocol and eluted in 30 μL of ddH_2_O.

### 2.4. Genotyping of the Pfmdr1 N86Y Mutation Using Restriction Fragment Length Polymorphism (RFLP)

Procedure described by Shrivastava et al. [4] was used for genotyping of the 86th codon. Five microliters (5 μL) from each of the purified nested amplicons was used in restriction digestion by incubating at 37 °C overnight using restriction enzymes *Afl*III (to detect mutational allele) and *Ap*oI (wild type allele). Enzymes were purchased from the New England Biolabs, UK. Then, 1 U of each enzyme, 2.5 μL of 10× NEB Buffer and 16.5 μL of nuclease free H_2_O was reconstituted for each sample (total reaction volume = 25 μL). The digests were resolved on 2% agarose gel stained with pEqGREEN, and the bands visualized.

### 2.5. Amplification, Cloning and Sequencing of Pfmdr1 Fragment Encompassing 86th and 184th Codons

Fragments of the *pfmdr1* gene encompassing the 86th and 184th codons were amplified in 16 DNA samples using previously described primers, F: 5′-AGAGAAAAAAGATGGTAACCTCA G-3′ and R: 5′-ACCACAAACATAAATTAACGG-3′ [39]. The reaction mixture comprises 1.6 µL of gDNA, 0.8 µL each of forward and reverse primers, 10 µL of GoTaq Green master mix (Promega, Madison, WI, USA) and 6.8 µL of ddH_2_O. The thermocycling condition was as follows: initial denaturation at 94 °C for 3 min, followed by 35 cycles each of 94 °C for 30 s, 60 °C for 1 min, 72 °C for 1 min and a final extension of 5 min at 72 °C. These PCR products were purified using QIAquick PCR Purification Kit, eluted in 30 μL of ddH_2_O and sequenced on both strands using the above primers. The purified products were ligated into pJET1.2 (CloneJET PCR Kit, ThermoFisher Scientific, Waltham, MA, USA), transformed into *DH5α E. coli* cells (Promega, Madision, WI, USA), PCR confirmed, and positive colonies (with *pfmdr1* fragment inserted in pJET1.2) were mini-prepped overnight. Details of the cloning protocol and sequencing were as performed for *pfkelch13* fragments in a previous study [22].

### 2.6. Amplification, Cloning and Sequencing of Pfcrt Fragment Encompassing the 76th Codon

Fragments of the *pfcrt* gene encompassing the 76th codon were amplified from 18 extracted DNA samples, successfully. A complete sequence of *pfcrt*, PF3D7_07909000, with accession number: KM288867.1 [40] was retrieved from GenBank. A pair of primers, (F: 5′-GATGGCTCACGTTTAGGTGGA-3′ and R: 5′-TGAATTTCCCTTTTTATTTCCAAA-3′), targeting exon-2 of the gene were designed to amplify a 267 bp fragment. A reaction mixture comprised 1.6 µL of gDNA, 0.8 µL each of forward and reverse primers, 10 µL of GoTaq Green master mix (Promega, USA) and 6.8 µL of ddH_2_O. The thermocycling condition was initial denaturation at 94 °C for 3 min, followed by 35 cycles, each at 94 °C for 30 s, 60 °C for 1 min, 72 °C for 45 s, and a final extension of 5 min at 72 °C. The purification, cloning and sequencing of these fragments was done as described above for the *pfmdr1* fragments.

### 2.7. Data Analysis

The haplotype frequency was calculated as the ratio of the sample(s) with the mutation to the total number of individual samples genotyped successfully. Polymorphism analysis was carried out through the manual examination of the sequence traces using Bioedit version 7.2.3.0 [35] and/or nucleotides/amino acid differences from multiple sequence alignments. Genetic parameters such as the number of haplotypes (h) and its diversity (H_d_), number of polymorphic sites (S) and nucleotide diversity (π) were computed using DnaSP 5.10 [37]. Different haplotypes were compared by constructing a maximum likelihood phylogenetic tree using MEGA 6.06 [38].

## 3. Results

### 3.1. Cytochrome Oxidase III PCR Confirmation of Infection

*The cytochrome oxidase* III PCR confirmed 90 out of 91 samples as *P. falciparum* positive with a band size of ~500 bp (Figure S1 shows 18 positive samples, together with a band for the positive control, PF3D7).

### 3.2. Amplification of Pfmdr1 Fragment Encompassing the 86th Codon and Genotyping

**Figure 1:**
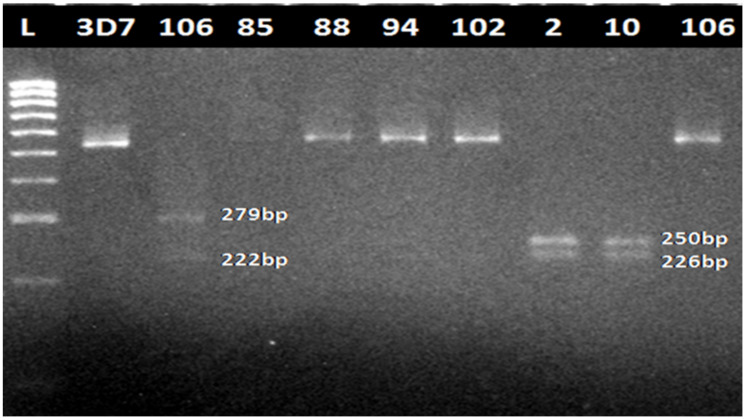
**Photomicrograph of agarose gel showing the results of restriction fragment length polymorphism (RFLP) analysis.** L is 100 bp Bioline DNA ladder [(100–1013 bp (40–200 ng/band)]; lane 2 is AflIII-negative (undigested) susceptible *PF3D7* sample, lane 3 is AflIII-positive sample 106 (chloroquine-resistant (CQR) isolate), samples 88, 94, 102 are AflIII-negative, samples 2 and 10 are ApoI-positive (chloroquine-sensitive (CQS) isolates), sample 106 is ApoI-negative. Samples were digested with restriction enzymes *Afl*III and *Apo*I.

A partial fragment of *pfmdr1* was amplified successfully from the above 90 DNA samples in the first-round PCR and 88 samples in the nested PCR (501 bp). All purified nested PCR products were subjected to restriction digestion with *Apo*I and *Afl*III. 87 (98.86%) samples were digested by *Apo*I (ApoI + ve) at position 86 giving three fragments of 250 bp, 226 bp and 25 bp, characteristic of chloroquine-sensitive isolates (CQS) (Figure 1). On digestion with *Afl*III, only one sample, MM106 (1.14%), was digested (AflIII + ve), giving two fragments of 279 and 222 bp consistent with chloroquine-resistant (CQR) isolates (86Y).

### 3.3. Polymorphism Analysis of Fragments of Pfmdr1 and Pfcrt

**Figure 2:**
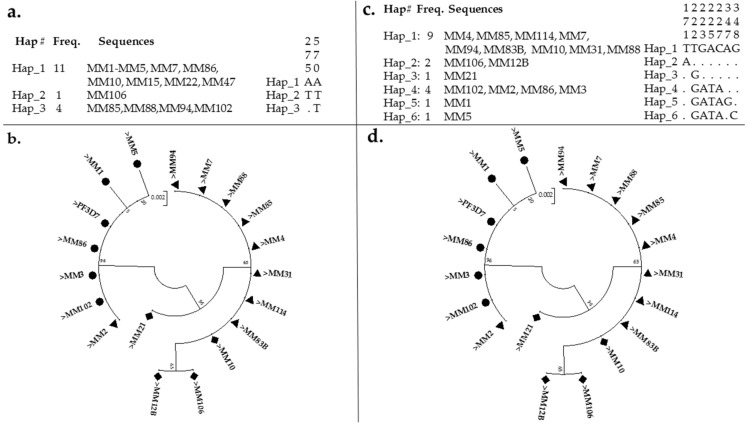
**Pattern of the genetic variability and polymorphism of *pfmdr1* and *pfcrt* fragments**: (**a**) showing haplotypes and their respective frequencies for *pfmdr1*, as well as polymorphic sites with respect to the major haplotype (Hap_1, inset); (**b**) a maximum likelihood phylogenetic tree of the pfmdr1 sequences. Haplotypes from mutated sequences are presented in a filled triangle, while that of MM106 (*pf mdr1* 86Y184F allele) is in diamond, while the rest of the sequences and *PF3D7* are presented in a filled cycle; (**c**) showing haplotypes and their respective frequencies for *pfcrt*, as well as polymorphic sites with respect to the major haplotype (Hap_1, inset); (**d**) a maximum likelihood phylogenetic tree of the *pfcrt* sequences. Haplotypes from mutated sequences are presented in the filled triangle, those of outliers MM106 and MM12B with additional mutation, as well as the MM21 with CVMET haplotype are in diamond, while the rest of the susceptible haplotypes and *PF3D7* are presented in filled cycle.

Analysis of a fragment of *pfmdr1* (610 bp encompassing the 86th and 184th codon) from 16 field sequences revealed a very low polymorphism (Figure S4 portrayed agarose gel picture). The sequences exhibited three haplotypes and two polymorphic sites both of which were non-synonymous, leading to amino acid substitutions (Figure 2a,b). Three sequences, MM85, MM88 and MM94 harbor the 184F mutation, and a fourth sequence MM106 is confirmed as a double mutant (86Y-184F).

This, in addition to a low haplotype diversity (H_d_ = 0.492) and nucleotide diversity of 0.00096, as well as the absence of synonymous mutations, suggest a high homogeneity in pfmdr1 from Kano isolates (Table 1). A neutrality test of all sequences revealed Tajima’s D and Li and Fu’s D * statistics as negative but not statistically significant.

**Figure 3:**
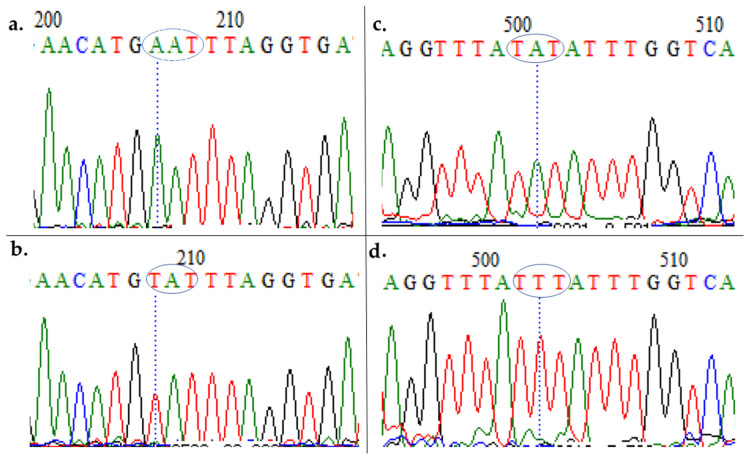
Analysis of the polymorphism of a fragment of the *pfmdr1* gene, spanning the 86th and 184th codons. Polymorphic codons for the 86th codon: (**a**) *PF3D7* and (**b**) MM106; for 184th codon: (**c**) *PF3D7* and (**d**) MM106.

Sequence analyses revealed the **A**AT->**T**AT polymorphism (Figure 2a and Figure 3a,b) in codon 86, led to the 86Y substitution in sample MM106 (Figure S2). This is the same AflIII positive sample from the restriction fragment length polymorphism (RFLP) (Figure 1). The rest of the field sequences were all AAT in this position, in complete agreement with the digestion results (above).

The Y184F substitution (T**A**T->T**T**T polymorphism) was also found in MM106 and four additional isolates (Figure 3c,d and Figure S2), leading to a frequency of 31.25%. Sequences cluster according to their haplotypes in the maximum likelihood phylogenetic tree (Figure 2b), with the sequences bearing mutations forming a cluster separate from the rest of sequences of the major haplotype with no mutation (which cluster together with the *PF3D7* sequence). These sequences have been deposited in GenBank with accession numbers MT438701–MT438717.

The analysis of a fragment of *pfcrt* (Figure S5 (267 bp encompassing the 72nd–76th codons)) from 18 field sequences revealed that 11 (61.11%) were CQR (CVIET, with two sequences MM106 and MM12B having an additional mutation in position 57, D^57^E), one sequence (5.55%) was CQR (CVMET), while six sequences (33.33%) were CQS (CVMNK). Two of these susceptible sequences, MM1 and MM5, have an additional mutation, K116R and K^116^N, respectively. Sample MM106 is the same double mutant, *pfmdr*86Y-184F, above. MM12B (*pfcrt*CVIET + D57E) and MM83B (*pfcrt*CVIET) are two samples from a previous study [22] which were found to harbor E^433^G and E^688^K *kelch13* mutations, respectively, in isolates from individuals that failed to clear infection 14 days following treatment with Cartef. In addition, MM21 (*pfcrt*CVMET), MM31 (CVIET) and MM114 (CVIET) are samples from [22] with *kelch13* F^434^I, F^434^I and F^434^S mutations in day 0 samples. The 18 sequences revealed a high polymorphism, exhibiting six haplotypes and seven polymorphic sites all of which led to amino acid substitutions (Figure 2c,d). This, in addition to a high haplotype diversity (H_d_ = 0.719) and nucleotide diversity of 0.00879, suggest a low homogeneity (Table 1). A neutrality test of all sequences revealed Tajima’s D and Li and Fu’s D* statistics as positive but not statistically significant. However, the presence of a predominant haplotype (Hap_1 made exclusively of CQR sequences, CVIET) suggests low diversity in the resistant isolates.

**Figure 4:**
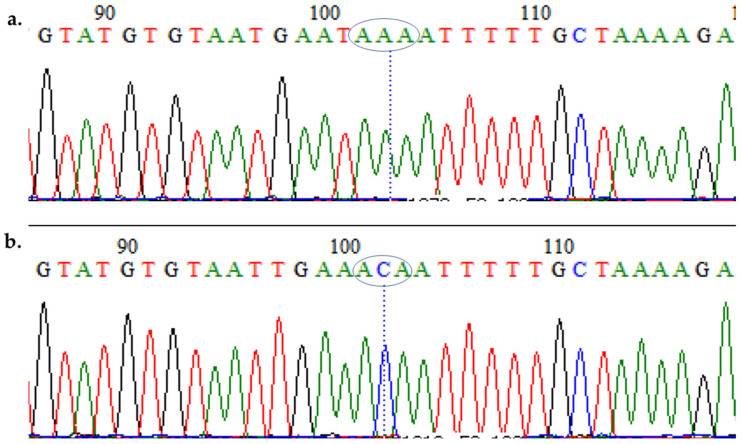
**Analysis of the polymorphism of a fragment of the *pfcrt* spanning the 76th codon.** Polymorphic codons for (**a**) CQS isolate bearing a CVMNK haplotype; and (**b**) CQR isolates bearing the CVIET haplotype.

The analysis of a DNA sequence confirmed ATG_74_->ATT, AAT_75_->GAA, and AAA_76_->ACA mutations discriminating C_72_V_73_M_74_N_75_K_76_ haplotypes from the evolved CQR haplotypes (C_72_V_73_I_74_E_75_T_76_) (Figure 4a,b and Figure S3). Figure S3 also presented the novel mutations in codon 116, which led to K^116^R and K^116^N in MM_1 and MM_5, respectively. Sequences cluster according to their haplotypes in the maximum likelihood phylogenetic tree (Figure 2d), with the CQR sequences forming a cluster separate from the CQS sequences, which in turn cluster together with the *PF3D7* sequence. These sequences were also deposited in GenBank with the accession numbers: MW267856–MW267873.

## 4. Discussion

Determination of the molecular markers of resistance helps in the epidemiological surveillance of antimalarial resistance in endemic regions, guiding respective malaria control programs to implement evidence-based control measures and resistance management. Widespread resistance necessitates frequent modifications of malaria treatment guidelines. As a first-line antimalarial drug, CQ has been dubbed the ‘*magic bullet*’, being used extensively for almost five decades, since it is cheap and highly effective [41]. However, the use of CQ was banned in several regions due to increasing chloroquine resistance [41]. This led to switching to SP, which met the same fate as CQ [42], though it is still used for the intermittent treatment of malaria in pregnant women (IpTP), and seasonal malaria chemoprevention (SMC) in areas with low resistance. However, the suspected failure of ACTs is leading to the increased use of CQs and other non-ACT antimalarials. In 2013, a former WHO country representative in Nigeria revealed that, “in spite of the ban placed on the use of CQ, artesunate (AS), SP, and other monotherapies, the drugs continue to thrive in Nigeria” [42]. This indiscriminate use of different antimalarials could select multi-resistance, especially in genes like the *pfmdr1* and *pfcrt* established to modulate resistance to various antimalarials.

### 4.1. Evidence of Resistance Haplotypes in pfmdr1 in P. falciparum Isolates from Kano

Genotyping the 86th codon and sequencing identified the 86Y mutation, which is known to mediate decreased susceptibility to CQ and amodiaquine, lumefantrine and dihydroartemisinin [29,43] in a single field isolate from Kano. The frequency of the 86Y mutation (1.14%) in this study is lower compared to the recent observations of Ikegbunam et al. [24] from southeast Nigeria (8.54%) and Muhammad et al. [23] in a study carried out with samples from Kano state (frequency of 86Y = 11.3%). However, a frequency of 62.2% was reported in a study from southwest Nigeria, suggesting that the frequency of this mutations varies from one region of Nigeria to another [44].

The presence of the 184F mutation has also been documented in Nigeria with high frequency. Examples include a frequency of 29.27% in southeast Nigeria [24], 28.1% in southwest Nigeria [25] and up to 69.0% in another study conducted in southwest Nigeria [44]. The 184F is said to have limited effect on its own, but its presence with N86 (N86+184F double mutant) [28] is known to be associated with reduced piperaquine susceptibility. Indeed, the presence of the 184F mutation mostly in combination with 86N was seen in reinfections after treatment with artemether–lumefantrine in a previous study [45].

In this study, the D1246Y mutation was not investigated since it is known to be present in approximately only 0.7–3% of the African isolates [10,30,31]. However, two studies have recorded 1246Y frequencies of 3.66 and 18.6%, respectively, in southeast and southwest Nigeria [24,30]. A recently published study conducted in Lagos, southwest Nigeria, detected rare *pfmdr1* mutations, N504K, N649D, F938Y and S967N, which were previously unreported [21], suggesting that the proactive surveillance of this gene and the validation of mutations discovered in it are necessary to ameliorate the menace of multidrug resistance. The occurrence of a high CQ susceptible allele with N86, as observed in this study, may be the result of the re-emergence of sensitive haplotypes or due to pressure from artemether–lumefantrine exposure [46]. Indeed, withdrawing chloroquine in a region where resistance is present could result in the re-emergence of CQ-sensitive parasites as shown in Malawi [47], which could also select for alternative resistance linked to the N86 allele, as explained above.

### 4.2. Evidence of Resistance Haplotypes in pfcrt in P. falciparum Isolates from Kano

The majority of effective mutations known to confer the CQR phenotype occur as a cluster between codons 72 and 76 [31,48] leading to the evolution of two major global CQR genotypes (CVIET and SVMNT) from the ancestral CVMNK haplotype [33]. The absence of the SVMNT haplotype in the isolates from Kano, consistent with the observations that this haplotype is predominant in the South American continent [31,33]. Indeed, recently published study on isolates from southeast Nigeria have shown a haplotypes distribution of 5.45, 0.00 and 76.37%, for CVMNK, SVMNT and CVIET, respectively [24]. Across southwestern Nigeria studies have documented 76T frequencies of 75.9% in isolates from Lagos [25], CVIET haplotype frequency of up to 91.6% for CVIET in a study conducted in Lagos [44], as well 60 and 63% 76T frequencies in isolates shown to confer resistance, respectively to CQ and amodiaquine [11,13]. In northern Nigeria, the frequency of 76T mutation (12.4%) described in northwest is much lower compared to 61.11% obtained in our study. Taken together, the high frequency of CQ-resistant haplotypes almost two decades after a change in antimalarial policy in Nigeria suggests the possibility of (i) the unofficial use of CQ which maintains the resistance alleles, and/or (ii) the exposure to structurally and/or functionally related antimalarials which are exerting selective pressure on this gene. Other factors including frequent travels known to introduce new strains and/or mutations, e.g., [49] cannot be ruled out.

## 5. Conclusions

This study established major mutations linked to antimalarial resistance in *pfmdr1* and *pfcrt* genes in *P. falciparum* isolates from Kano, northwestern Nigeria. The spread of these mutations should be monitored as these genes are known to modulate resistance to various antimalarials currently used in northern Nigeria; it should be considered while designing control measures and/or resistance management approaches. More research needs to be carried out on these genes, for example, (i) the investigation of the presence of 184F and 1246Y mutations using more samples; (ii) qRT-PCR of *pfmdr1* to detect potential copy number variations, known to be associated with resistance to various antimalarials, including mefloquine, lumefantrine, quinine and artemisinin [29]; the validation of the role of the *pfcrt* D57E additional mutation discovered in two CVIET sequences, using in vitro assays; as well as (iii) the sequencing of more partial fragments or full *pfcrt* from field samples to detect and validate more mutations.

## Figures and Tables

**Table 1:** Summary statistics for the polymorphism of *pfmdr1* and *pfcrt* fragments.

Location	N	S	H	H_d_	Syn	Nonsyn	π (k)	D (Tajima)	D* (Fu and Li)
						***pfmdr1***			
Kano	16	2	3	0.49	0	2	0.00096 (0.583)	−0.0823 ^ns^	−0.5038 ^ns^
						***pfcrt***			
Kano	18	7	6	0.72	0	7	0.00879 (2.346)	0.518 ^ns^	0.0939 ^ns^

N; number of sequences; S, number of polymorphic sites; h, haplotype; H_d_, haplotype diversity; Syn, synonymous mutations; Nonsyn, non-synonymous mutations; π, nucleotide diversity (k = mean number of nucleotide differences); Tajima’s D and Fu and Li’s D* statistics; ns, not significant.

## Data Availability

The DNA sequences reported in this study have been deposited in the GenBank with accession numbers of MT438701–MT438717 for *pfmdr1* and MW267856–MW267873 for *pfcrt*, respectively.

## Supplementary Materials

The following are available online at https://www.mdpi.com/2079-9721/9/1/6/s1, Figure S1: **Photomicrograph of agarose gel for *COX* III PCR.** HL is a DNA ladder (hyperladder 100 bp, Bioline, 100–1013 bp); lanes 1–18 shows a band of approximately 500 bp characteristics of *COX* III fragment of *P. falciparum*; lane 19 is for *PF3D7* positive control gDNA, Figure S2: **Comparison of sequences of *pfmdr1* of field isolates and PF*3D7.*** Residues affected by mutations are highlighted in grey, with isolate MM106 exhibiting double mutations, 86Y and 184F, Figure S3: **Comparison of sequences of *pfCRT* of field isolates and PF*3D7*.** Residues affected by mutations are highlighted in grey, with MM106 and MM12B exhibiting D57E mutation, in addition to being a CVIET haplotype. Figure S4: **Photomicrograph of agarose gel for *pfmdr1*.** L is a DNA ladder (hyperladder 100 bp, Bioline, 100–1013 bp); lane 2 is PF3D7 positive control gDNA, and 3–18 show bands of approximately 610 bp, characteristic of the pfmdr1 fragment encompassing the 86th and 184th codons, Figure S5: **Photomicrograph of agarose gel for *pfcrt*.** L is a DNA ladder (hyperladder 100 bp, Bioline, 100–1013 bp); lanes 2–19 is 267 bp *pfcrt* fragments encompassing codons 72–76; lane 20 is for PF3D7 positive control gDNA.
