# Supplementary material for: Polymorphism Analysis of pfmdr1 and pfcrt from Plasmodium falciparum Isolates in Northwestern Nigeria Revealed the Major Markers Associated with Antimalarial Resistance
Source: Diseases. 2021 Jan 4;9(1):6. doi: 10.3390/diseases9010006 (PMC7838797; doi:10.3390/diseases9010006)
Supplement: Supplementary file 1 [file diseases-09-00006-s001.pdf]

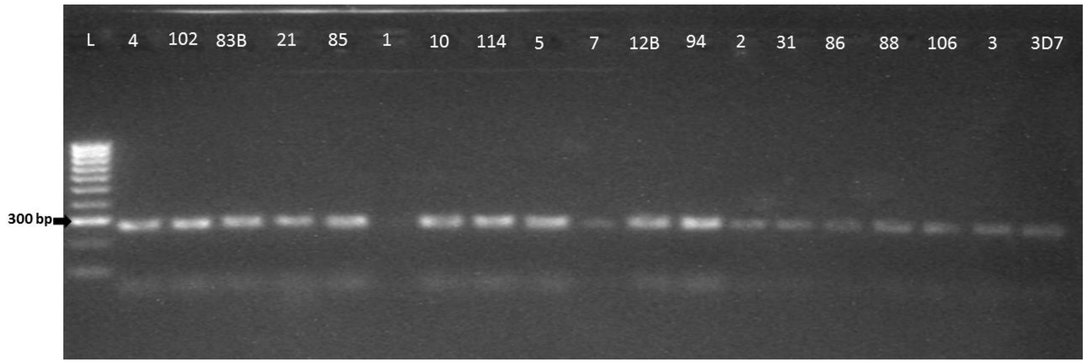

Figure S1: **Photomicrograph of agarose gel for *COX* III PCR.** HL is a DNA ladder (hyperladder 100 bp, Bioline, 100–1013 bp); lanes 1–18 shows a band of approximately 500 bp characteristics of *COX* III fragment of *P. falciparum*; lane 19 is for *PF3D7* positive control gDNA

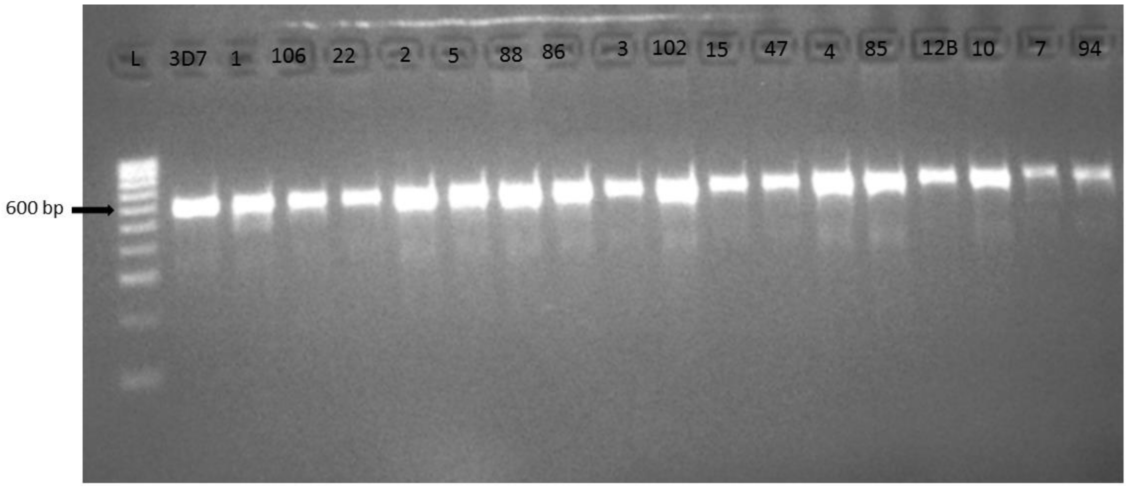

Figure S2: **Comparison of sequences of *pfmdr1* of field isolates and PF*3D7*.** Residues affected by mutations are highlighted in grey, with isolate MM106 exhibiting double mutations, 86Y and 184F

```
                                   20                                 40
                                   |                                  |
>pf3D7_CRT   NGSRLGGGSC  LGKCAHVFKL  IFKEIKDNIF  IYILSIIYLS  VCVMNKIFAK  50
>MM106_CRT   NGSRLGGGSC  LGKCAHVFKL  IFKEIKENIF  IYILSIIYLS  VCVIETIFAK  50
>MM12B_CRT   NGSRLGGGSC  LGKCAHVFKL  IFKEIKENIF  IYILSIIYLS  VCVIETIFAK  50
>MM21_CRT    NGSRLGGGSC  LGKCAHVFKL  IFKEIKDNIF  IYILSIIYLS  VCVMETIFAK  50
>MM1_CRT     NGSRLGGGSC  LGKCAHVFKL  IFKEIKDNIF  IYILSIIYLS  VCVMNKIFAK  50
>MM5_CRT     NGSRLGGGSC  LGKCAHVFKL  IFKEIKDNIF  IYILSIIYLS  VCVMNKIFAK  50

                      60                                 80
                      |                                  |
>pf3D7_CRT   RTLNKIGNYS  FVTSETHNFI  CMIMFFIVYS  LFGNKKGNS  89
>MM106_CRT   RTLNKIGNYS  FVTSETHNFI  CMIMFFIVYS  LFGNKKGNS  89
>MM12B_CRT   RTLNKIGNYS  FVTSETHNFI  CMIMFFIVYS  LFGNKKGNS  89
>MM21_CRT    RTLNKIGNYS  FVTSETHNFI  CMIMFFIVYS  LFGNKKGNS  89
>MM1_CRT     RTLNKIGNYS  FVTSETHNFI  CMIMFFIVYS  LFGNKRGNS  89
>MM5_CRT     RTLNKIGNYS  FVTSETHNFI  CMIMFFIVYS  LFGNKNGNS  89
```

Figure S3: **Comparison of sequences of *pfCRT* of field isolates and PF*3D7*.** Residues affected by mutations are highlighted in grey, with MM106 and MM12B exhibiting D57E mutation, in addition

to being a CVIET haplotype.

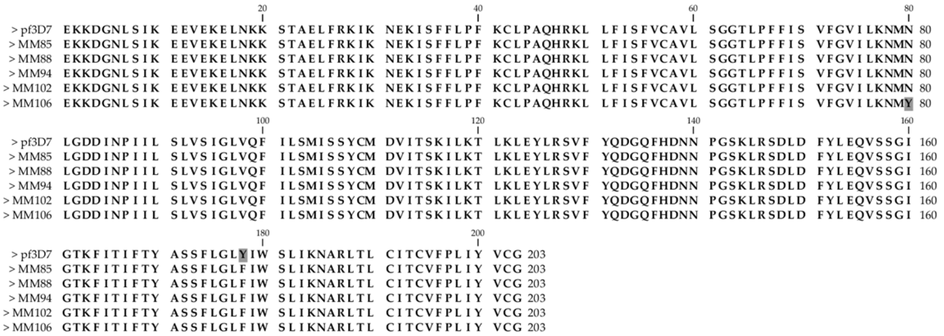

Figure S4**: Photomicrograph of agarose gel for *pfmdr1*.** L is a DNA ladder (hyperladder 100 bp, Bioline, 100–1013 bp); lane 2 is PF3D7 positive control gDNA, and 3–18 show bands of approximately 610 bp, characteristic of the pfmdr1 fragment encompassing the 86th and 184th codons.

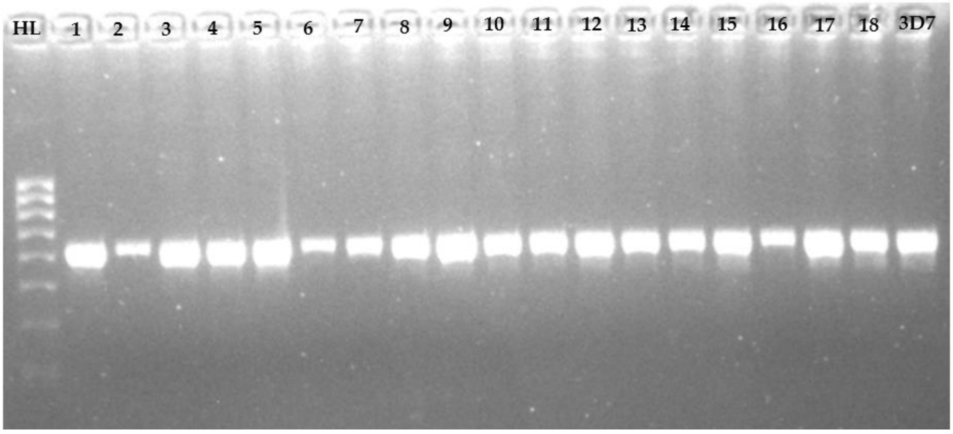

Figure S5: **Photomicrograph of agarose gel for *pfcrt*.** L is a DNA ladder (hyperladder 100 bp, Bioline, 100–1013 bp); lanes 2–19 are 267 bp *pfcrt* fragments encompassing codons 72–76; lane 20 is for PF3D7 positive control gDNA.
